# Caffeine Produced in Rice Plants Provides Tolerance to Water-Deficit Stress

**DOI:** 10.3390/antiox12111984

**Published:** 2023-11-08

**Authors:** Youngchul Yoo, Yo-Han Yoo, Dong Yoon Lee, Ki-Hong Jung, Sang-Won Lee, Jong-Chan Park

**Affiliations:** 1 Advanced Radiation Technology Institute (ARTI), Korea Atomic Energy Research Institute (KAERI), Jeongeup 56212, Republic of Korea; yooyc84@kakao.com; 2 Central Area Crop Breeding Division, Department of Central Area Crop Science, National Institute of Crop Science, RDA, Suwon 16429, Republic of Korea; yohan04@korea.kr; 3 Graduate School of Green-Bio Science, Crop Biotech Institute, Kyung Hee University, Yongin 17104, Republic of Korea; ehddbs0502@khu.ac.kr (D.Y.L.); khjung2010@khu.ac.kr (K.-H.J.); 4 Plant Systems Engineering Research Center, Korea Research Institute of Bioscience & Biotechnology (KRIBB), Daejeon 34141, Republic of Korea

**Keywords:** caffeine, water-deficit stress tolerance, calcium-dependent protein kinase, antioxidants, transcription factors

## Abstract

Exogenous or endogenous caffeine application confers resistance to diverse biotic stresses in plants. In this study, we demonstrate that endogenous caffeine in caffeine-producing rice (CPR) increases tolerance even to abiotic stresses such as water deficit. Caffeine produced by CPR plants influences the cytosolic Ca^2+^ ion concentration gradient. We focused on examining the expression of Ca^2+^-dependent protein kinase genes, a subset of the numerous proteins engaged in abiotic stress signaling. Under normal conditions, CPR plants exhibited increased expressions of seven *OsCPKs* (*OsCPK10*, *OsCPK12*, *OsCPK21*, *OsCPK25*, *OsCPK26*, *OsCPK30*, and *OsCPK31*) and biochemical modifications, including antioxidant enzyme (superoxide dismutase, catalase, peroxidase, and ascorbate peroxidase) activity and non-enzymatic antioxidant (ascorbic acid) content. CPR plants exhibited more pronounced gene expression changes and biochemical alterations in response to water-deficit stress. CPR plants revealed increased expressions of 16 *OsCPKs* (*OsCPK1*, *OsCPK2*, *OsCPK3*, *OsCPK4*, *OsCPK5*, *OsCPK6*, *OsCPK9*, *OsCPK10*, *OsCPK11*, *OsCPK12*, *OsCPK14*, *OsCPK16*, *OsCPK18*, *OsCPK22*, *OsCPK24*, and *OsCPK25*) and 8 genes (*OsbZIP72*, *OsLEA25*, *OsNHX1*, *OsRab16d*, *OsDREB2B*, *OsNAC45*, *OsP5CS*, and *OsRSUS1*) encoding factors related to abiotic stress tolerance. The activity of antioxidant enzymes increased, and non-enzymatic antioxidants accumulated. In addition, a decrease in reactive oxygen species, an accumulation of malondialdehyde, and physiological alterations such as the inhibition of chlorophyll degradation and the protection of photosynthetic machinery were observed. Our results suggest that caffeine is a natural chemical that increases the potential ability of rice to cope with water-deficit stress and provides robust resistance by activating a rapid and comprehensive resistance mechanism in the case of water-deficit stress. The discovery, furthermore, presents a new approach for enhancing crop tolerance to abiotic stress, including water deficit, via the utilization of a specific natural agent.

## 1. Introduction

Throughout their life cycle, plants encounter a broad spectrum of biotic and abiotic stresses. Among these challenges, drought stress emerges as a particularly formidable and pressing issue, primarily due to its extensive occurrence on a significant portion of the world’s land [1,2]. This widespread phenomenon is largely attributed to the recent and rapid changes in our climate [3,4]. Drought stress exerts a profound influence on the physiological, metabolic, and molecular functions of crops, ultimately leading to substantial reductions in crop yields [5,6,7]. This, in turn, poses a significant threat to global food security. Of particular concern is rice, the second most widely cultivated food crop globally, following maize [8,9,10]. The centrality of rice in the global food supply underscores its significance. Nevertheless, the water-intensive cultivation methods employed for rice make it especially vulnerable to water-deficit stress, as it necessitates two to three times more water for cultivation compared to other crops [11,12,13,14]. Consequently, there is a pressing need to accelerate the development of water-deficit-tolerant rice varieties and innovative technologies via a multifaceted approach.

Plants have developed defense mechanisms against biotic and abiotic stresses. One of these defense strategies involves the production of secondary metabolites that can directly or indirectly counteract the stressor [15,16]. An illustrative example of this mechanism is the accumulation of secondary metabolites, such as lignin. Numerous studies have provided evidence that certain genes, including *OsCCR10* [17] and *OsTF1L* [18] in rice, *PeLAC10* [19] in Arabidopsis, and *PoCCoAOMT* [20] in tobacco, play pivotal roles in enhancing drought tolerance by regulating the accumulation of lignin in response to drought stress. To date, an extensive array of over 200,000 secondary metabolites, spanning diverse biosynthetic families such as alkaloids, terpenes, and phenolics, have been identified in various plant species [15,21]. Caffeine (1, 3, 7-trimethylxanthine) is a major secondary metabolite of the purine alkaloid family and is synthesized in a few plant species, including coffee, cacao, and tea [22]. Although it is a very familiar compound now widely consumed in beverages such as coffee and tea, it originally played an important role in plants. Caffeine has allelopathic effects on other plants, where it can inhibit their growth and development, helping the caffeine-producing plant to gain an advantage in competing for resources [23]. Furthermore, several studies have shown that caffeine plays a role in protecting host plants from pathogens and herbivores, either directly or indirectly [22]. Such properties of caffeine have been employed in transgenic plants. Transgenic tobacco plants that synthesize caffeine are highly resistant to fungal pathogens, viruses, and herbivores [24,25,26,27,28]. In our previous study, we demonstrated that caffeine synthesized in transgenic rice (CPR, caffeine-producing rice) directly inhibits the activity of phosphodiesterase, resulting in an influx of calcium ions (Ca^2+^) into the cell. This causes alterations in the Ca^2+^ signals, which eventually drive the synthesis and accumulation of salicylic acid, providing resistance to diverse biotic stresses [27].

Ca^2+^ is a crucial element for plant growth and development [29,30]. It is essential for maintaining cell wall structure, cell division, and elongation [31]. Furthermore, changes in cytosolic free calcium concentration are among the swiftest intracellular responses to both abiotic and biotic stresses [32,33,34]. This transient increase in cytosolic calcium ion concentration generates a specific calcium signature, which can be detected by various calcium sensors, thereby initiating downstream effects [33,34]. Representatively, Ca^2+^-dependent protein kinases (CPKs) and calcium/calmodulin-regulated kinases (CAMKs) are activated via specific calcium signatures, subsequently triggering changes in gene expression patterns, production of reactive oxygen species (ROS), and the reinforcement of cell walls [35]. Nevertheless, excessive fluctuations in calcium levels can trigger programmed cell death or apoptosis within the hypersensitive response (HR) [36,37,38]. In our previous study, despite the production of caffeine in rice resulting in intracellular calcium changes, we did not observe severe HR responses or programmed cell death, even when the plants were subjected to various biotic stresses [27]. This phenomenon has not been previously reported in either rice or transgenic plants producing caffeine [25,26]. The reactions triggered by changes in intracellular Ca^2+^ concentration are also deeply involved in the response to abiotic stresses such as salinity, drought, cold, and heat [39,40,41]. Among them, drought stress impacts plant photosynthesis and overall ROS production via the signaling of the plant hormone abscisic acid (ABA), resulting in ROS accumulation and oxidative stress [42,43,44]. ROS molecules, previously believed only to have detrimental effects, are essential for long-distance signaling, response to stress, and the growth and development of plants [45]. However, the excessive production of ROS, on the other hand, can cause oxidative damage to essential cellular components such as nucleic acids, proteins, and cell membranes. Hence, the balance between ROS generation and detoxification is strictly controlled [46]. Changes in cytosolic Ca^2+^ concentration caused by water deficits play a role in the drought stress response mechanism in an ABA-dependent manner by activating CPKs and releasing ABA from the cell [47]. These mechanisms encompass cellular functions, including stomatal closure [48]. The ABA signals induce the activity and accumulation of enzymatic antioxidants such as superoxide dismutase (SOD), catalase (CAT), peroxidase (POX), and ascorbate peroxidase (APX), as well as non-enzymatic antioxidants such as ascorbic acid, carotenoids, and glutathione [49,50,51]. In addition, the expression of numerous genes is affected under drought stress, many of which play important roles in other abiotic stress responses and tolerance. Several genes with expressions induced under drought stress are also induced under salt stress, indicating a significant interaction between these two stresses [52,53].

We hypothesized that caffeine synthesized in CPR not only confers resistance to biotic stresses, as shown in our previous report [27], but may also contribute to providing tolerance to abiotic stresses, including water deficit. To prove this, the present study aimed to assess the efficacy of caffeine produced in transgenic rice as a tolerance-inducing agent to water-deficit stress and elucidate the mechanism underlying its stress tolerance properties. Our results indicated involvement in the expression changes in OsCPKs and transcription factors related to stress tolerance, leading to the enforcement of ROS detoxification and ultimately maintaining the stability of physiological factors related to photosynthesis. Our finding indicates that caffeine possesses the ability to enhance and augment resistance to water deficit, providing novel perspectives on strategies for developing tolerance to abiotic stresses.

## 2. Materials and Methods

### 2.1. Plant Growth Conditions and Water-Deficit Stress Treatment

Wild-type rice (WT, *Oryza sativa*. *japonica* cv. Dongjin) and two homozygous T_5_ transgenic rice lines (caffeine-producing transgenic rice, CPR #1 and CPR #2) were used in these experiments. Wild-type rice was obtained from the Institute of Crop Biotechnology at Kyung Hee University in Yongin, Republic of Korea. To generate the CPR lines, the full-length coding sequence (CDS) of *CaMXMT1* (AB048794), *CaXMT1* (AB048793), and *CaDXMT1* (AB084125) from *Coffea arabica* were amplified and cloned into vector pBIN-NMT 777 under the control of the 35s promoter. The constructed vector pBIN-NMT 777 was introduced into rice (Dongjin) using the Agrobacterium-mediated early infection method [54]. The detailed methodology has been previously reported [27]. Wild-type and transgenic rice plants were grown in a growth chamber (28 °C, 60% relative humidity, 14/10 h light/dark photoperiod, light intensity of 200 µmol m^−2^ s^−1^) for 28 days.

To minimize the potential variations in stress effects, we cultivated both wild-type and transgenic rice plants in the same pots. Each pot contained five wild-type and five CPR plants. Stress treatments were conducted on a total of nine wild-type/CPR1 pots and nine wild-type/CPR2 pots to ensure biological replicates. Wild-type and transgenic rice plants were grown in a growth chamber with normal watering for 28 days. Subsequently, they were transferred to a greenhouse, where water supply was then stopped for 10 days. Following the conclusion of the water-deficit stress treatment, water was re-provided, and the phenotypic analysis was monitored for 14 days. We employed a temperature and humidity data logger, RC-4HC (Elitech Technology, San Jose, CA, USA), to measure soil moisture, providing confirmation of the water-deficit stress. Samples for gene transcript expression analyses and antioxidant enzyme activity assays were collected every 2 days over the course of the 10-day water-deficit treatment.

### 2.2. Determination of Caffeine Contents in Rice Leaves

To extract caffeine from transgenic rice leaves, 1 g of the sample was homogenized in a 50% methanol solution and incubated with stirring at 60 °C for 1 h. The extract was then centrifuged at 14,000× *g* for 10 min at 4 °C. An HPLC system (Agilent Technologies 1200 series, CA, USA) equipped with a reverse-phase column (Eclipse XDB-C18 column, 4.6 mm × 150 mm, 5 µm; Agilent Technologies, Santa Clara, CA, USA) was used to separate caffeine. The ChemStation software ver. B.04.03 (Agilent Technologies, CA, USA) was used to calculate the results on an HPLC system equipped with a UV-VIS spectrophotometer. The mobile phase A consisted of trifluoroacetic acid and water (0.05:99.95, *v*/*v*), and the mobile phase B was acetonitrile. The gradient elution profile was as follows: 0–21 min, a linear gradient from 2% B to 9% B; 21–32 min, a linear gradient from 9% B to 23% B; 32–45 min, 23% B. The elution was carried out at a flow rate of 0.8 mL min^−1^. The caffeine peaks were identified by comparing their retention times with caffeine standards (Agilent Technologies, USA) at 280 nm. Standard curves for quantification were constructed using the quadratic fitting of the relationship between the area sum and the concentration of the peaks corresponding to each sample.

### 2.3. RNA Isolation and Quantitative Real-Time PCR (qRT-PCR) Analysis

Total RNA was extracted from rice leaf samples using RNAiso (Takara, Kusatsu, Japan) following the manufacturer’s instructions. DNase I treatment (Qiagen, Hilden, Germany) was performed to remove any residual DNA. The isolated RNA was quantified using a NanoDrop ND-1000 spectrophotometer (Thermo Fisher Scientific, Waltham, MA, USA). First-strand complementary DNA (cDNA) was synthesized from 2 µg of total RNA using the SuPrime-Script cDNA Synthesis Kit (GeNet Bio, Daejeon, Republic of Korea) according to the manufacturer’s instructions. The qRT-PCR was performed using 2X Prime Q-Mastermix SYBR Green I (GeNet Bio, Republic of Korea) reagent on a Rotor-Gene Q instrument system (Qiagen, Germany). The 2^−ΔΔCt^ method [55] was used to determine the relative quantification of gene expression. The fold change in gene expression was normalized to the internal control, *OsUBQ1* (AK121590, Os03g13170), and converted to the fold change using the data from the wild-type. The experiment was performed with three biological replicates. The primers used in the experiment are listed in Table S1.

### 2.4. Determination of Lipid Peroxidation and Reactive Oxygen Species Levels

Malondialdehyde (MDA) levels in rice leaf samples were determined using the Lipid Peroxidation (MDA) Assay Kit (Sigma-Aldrich, St. Louis, MO, USA) according to the manufacturer’s instructions. Ten milligrams of rice leaves were homogenized with MDA lysis buffer and centrifuged at 13,000× *g* for 10 min, and the residue was discarded. To 200 µL of the sample, 600 µL of thiobarbituric acid was added and incubated at 95 °C for 1 h, followed by cooling on ice for 10 min. The MDA content in each sample was quantified as absorbance at 532 nm using the equation derived from the MDA standard curve. To determine the H_2_O_2_ content, 1 g of rice leaf samples was homogenized in 3 mL of 100 mM sodium phosphate buffer (pH 6.8). The homogenate was filtered through four layers of cheesecloth and centrifuged at 14,000× *g* for 20 min at 4 °C. The H_2_O_2_ content of the supernatant was measured via the modified Bernt and Bergmeyer [56] method using peroxidase. For this, 0.5 mL of supernatant was mixed with 2.5 mL of peroxide solution containing 83 mM sodium phosphate (pH 7.0), 0.005% *w*/*v* o-dianisidine, and 40 mg of peroxidase and incubated at 30 °C for 10 min. The reaction was terminated by the addition of 0.5 mL of 1 N perchloric acid, and the sample was then centrifuged at 5000× *g* for 5 min. The amount of H_2_O_2_ in each sample was quantified as the absorbance at 436 nm based on the equation derived from the H_2_O_2_ standard curve.

### 2.5. Activity Assays of Antioxidant Enzyme

For the determination of superoxide dismutase (SOD, EC: 1.15.1.1), catalase (CAT, EC: 1.11.1.6), and peroxidase (POX, EC: 1.11.1.7) activities, total proteins were extracted from 1 g of rice leaves via homogenization in 100 mM potassium phosphate buffer (pH 7.8) containing 0.1 mM EDTA, 1% *w*/*v* polyvinyl-pyrrolidone, and 0.5% *v*/*v* Triton X-100 at 4 °C. For the assay of ascorbate peroxidase (APX, EC: 1.11.1.11) activity, 1 g of rice leaves was homogenized in 100 mM sodium phosphate buffer (pH 7.0) containing 5 mM ascorbate and 1 mM EDTA. The homogenate was filtered through four layers of cheesecloth and centrifuged at 14,000× *g* at 4 °C for 25 min. The total protein content was determined according to the Bradford method [57]. The activity of SOD was determined according to the method of Beyer and Fridovich [58]. The reaction mixture consisted of 50 mM potassium phosphate (pH 7.8), 9.9 mM methionine, 57 µM nitro blue tetrazolium (NBT), and 40 µL protein extract. The reaction was initiated via light illumination. One unit of SOD is defined as the amount of enzyme that causes 50% inhibition of SOD-inhibitable NBT reduction. CAT activity was determined from the rate of H_2_O_2_ decomposition (extinction coefficient 39.4 mM/L cm^−1^), measured as the decrease in absorbance at 240 nm, according to the method of Aebi [59]. The reaction mixture contained 50 mM potassium phosphate buffer (pH 7.0) and 40 µL of the protein extract. The reaction was initiated by the addition of 10 mM H_2_O_2_. Peroxidase activity was determined by monitoring the formation of guaiacol dehydrogenation product (extinction coefficient 6.39 mM/L cm^−1^) at 436 nm according to the Pütter method [60]. The reaction mixture contained 100 mM potassium phosphate (pH 7.0), 0.3 mM guaiacol, and 50 µL protein extract. The reaction was initiated by the addition of 0.1 mM H_2_O_2_. APX activity was determined by measuring the decrease in absorbance at 290 nm (extinction coefficient 2.8 mM cm^−1^) according to the method of Chen and Asada [61]. The reaction mixture contained 50 mM potassium phosphate (pH 7.0), 0.5 mM ascorbate, 0.2 mM H_2_O_2_, and 10 µL protein extract.

### 2.6. Measurement of Chlorophyll, Carotenoid, Ascorbic Acid, and Proline Contents

Chlorophyll and carotenoid contents were determined using the method described by Lichtenthaler and Wellburn [62]. Freshly collected leaves (0.1 g) were homogenized with 80% (*v*/*v*) chilled acetone and centrifuged at 10,000× *g* at 4 °C for 10 min. After filtration, the optical density was measured using a UV-1800 spectrophotometer (Shimadzu, Kyoto, Japan) at 663, 645, and 440 nm. The pigment levels were calculated using the following equations:

Total chlorophyll (mg g^−1^) = 20.2 × Abs_645_ + 8.05 × Abs_663_

Chlorophyll a (mg g^−1^) = 12.7 × Abs_663_ − 2.69 × Abs_645_

Chlorophyll b (mg g^−1^) = 22.9 × Abs_645_ − 4.68 × Abs_663_

Carotenoids (mg g^−1^) = 4.7 × Abs_440_ − (1.38 × Abs_663_ + 2.69 × Abs_645_)

The ascorbic acid content of the plants was measured using the protocol of Mukherjee and Choudhuri [63]. A sample of 0.5 g of fresh leaves was homogenized with 10 mL of trichloroacetic acid and then filtered. One drop of 10% thiourea, 2 mL of 2% diphenylhydrazine, and 5 mL of 80% H_2_SO_4_ were added to 4 mL of the supernatant of the plant leaf extract in test tubes. Absorbance was measured at 530 nm using a UV-1800 spectrophotometer. The ascorbic acid content in each sample was quantified as the absorbance at 530 nm using the equation obtained from the ascorbic acid standard curve.

The proline content of the plant sample was determined using the method of Bates et al. [64]. Half a gram of fresh leaf sample was homogenized in 5 mL of 3% sulphosalicylic acid, filtered, and centrifuged at 10,000× *g* for 15 min. The collected 2 mL of supernatant was mixed with 2 mL of acid ninhydrin and 2 mL of glacial acetic acid. These test tubes were placed in a water bath at 80 °C for 1 h. The tubes were then kept in an ice bath to terminate the reaction. The developed chromophore was extracted with 4 mL of toluene. Using the equation from the proline standard curve, the proline content in each red-colored solution was quantified as the absorbance at 520 nm.

### 2.7. Measurement of Transient Chlorophyll a Fluorescence

Chlorophyll fluorescence was measured using a HANDY PEA fluorimeter (Hansatech, King’s Lynn, London, UK) on the longest leaves at their apex, midsection, and base. Prior to all measurements, the leaves were dark-adapted for a period of 30 min. The light intensity was 3500 µmol photons m^−2^ s^−1^, provided by an array of three high-intensity light-emitting diodes, focused on a spot with a diameter of 5 mm. The measurements were recorded for 1 s at a resolution of 12-bits. The fluorescence parameters Fv/Fm were obtained using HANDY PEA software (version 1.31).

### 2.8. Statistical Analysis

All data are presented as mean ± standard deviation, as indicated in each figure. Student’s *t*-test (** *p* < 0.01) was used to compare each data point with the control to test for significant differences. In addition, one-way analysis of variance (ANOVA) followed by Duncan’s test was used to statistically analyze differences between data points. Statistical significance was set at *p* < 0.01 using SPSS software ver. 22.0 (IBM, New York, NY, USA).

## 3. Results

### 3.1. Endogenous Caffeine in CPR Plants Alters OsCPKs Expression

**Figure 1:**
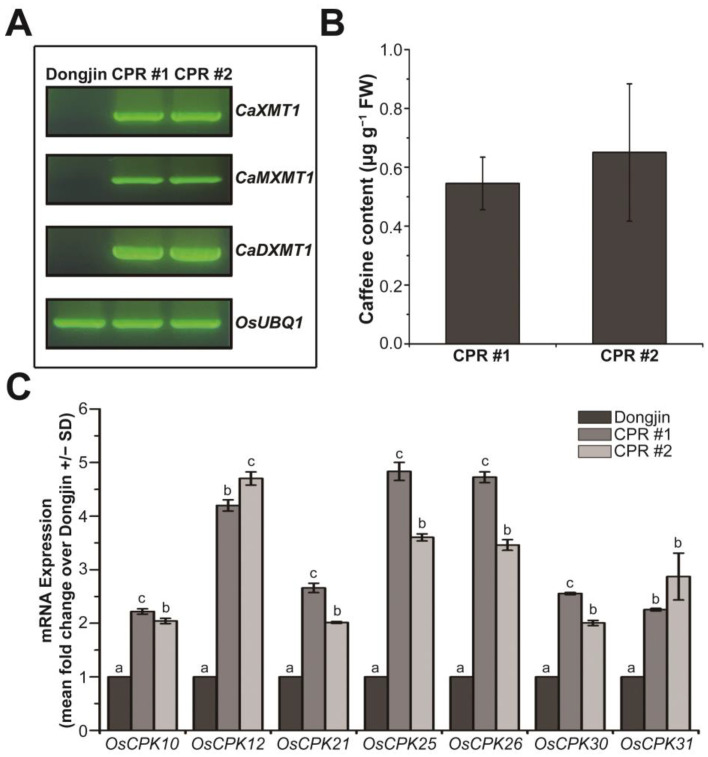
Caffeine synthesis and expression of *OsCPKs* in caffeine-producing transgenic rice (CPR, transgenic CPR line #1 and #2) and wild-type plants (Dongjin) at four-leaf stage under normal conditions. (**A**) Validation of the expression of the introduced *CaMXMT1*, *CaXMT1*, and *CaDXMT1* genes in CPR plants. Total RNA was extracted from leaves of wild-type and CPR plants at the four-leaf stage, and reverse transcription-polymerase chain reaction (RT-PCR) was performed using specific primers for each of the three introduced genes. (**B**) Validation of caffeine synthesis in leaves of CPR plants at the four-leaf stage. Caffeine was extracted from CPR plant leaves by methanol extraction and detected by high-performance liquid chromatography (HPLC). All experiments were repeated three times. Error bars represent the means ± SD (*n* = 3). (**C**) Expression of *OsCPKs* gene in Dongjin and CPR plants at the four-leaf stage under normal conditions. Total RNA was extracted from leaves of wild-type and CPR plants at the four-leaf stage, and quantitative RT-PCR (qRT-PCR) was performed using specific primers for each *OsCPK* gene. All experiments were repeated three times. Error bars represent the means ± SD (*n* = 3). Different letters above bars indicate statistically significant differences as determined by one-way analysis of variance (ANOVA), *p* < 0.01.

In our previous study, transgenic rice plants producing caffeine were prepared by introducing three N-methyltransferase genes for caffeine synthesis into rice plants, and we investigated the role of endogenous caffeine in response to various biotic stresses [27]. In this study, we used the transgenic rice plants of the T_5_ generation (the next generation of the plants that we used for the previous study), specifically the independent transgenic lines CPR #1 and CPR #2, and confirmed the expression of the introduced genes via RT-PCR (Figure 1A). Using HPLC, we detected the accumulation of caffeine with a content of approximately 0.545 µg g^−1^ fresh weight in CPR #1 and 0.65 µg g^−1^ fresh weight in CPR #2 at the four-leaf stage (Figure 1B), which was similar to the T_4_ generation of CPR plants [27], indicating stable caffeine synthesis across generations. In CPR plants, the production of caffeine via a series of processes leads to cytosolic calcium accumulation, resulting in changes in the expression of calcium sensors, CPKs, which have various effects on calcium signaling [27]. We hypothesized that even though the synthesis of caffeine is relatively small at this four-leaf stage, it would have a significant effect on CPK expression. To test this, we analyzed the expression of 31 *OsCPKs* via qRT-PCR and found that *OsCPK10* (LOC_Os03g57450), *OsCPK12* (LOC_Os04g47300), *OsCPK21* (LOC_Os08g42750), *OsCPK25* (LOC_Os11g04170), *OsCPK26* (LOC_Os12g03970), *OsCPK30* (LOC_Os07g44710), and *OsCPK31* (AK110341) were more than 2-fold upregulated in CPR plants at the four-leaf stage compared with wild-type plants (Figure 1C). Other *OsCPKs* were either similarly expressed or downregulated in CPR plants compared to wild-type plants (Figure S1). Those *OsCPKs* are well known as positive regulators that enhance tolerance to abiotic stresses as well as to biotic stresses [65,66,67,68,69,70].

### 3.2. Endogenous Caffeine in CPR Plants Confers Water-Deficit Tolerance

**Figure 2:**
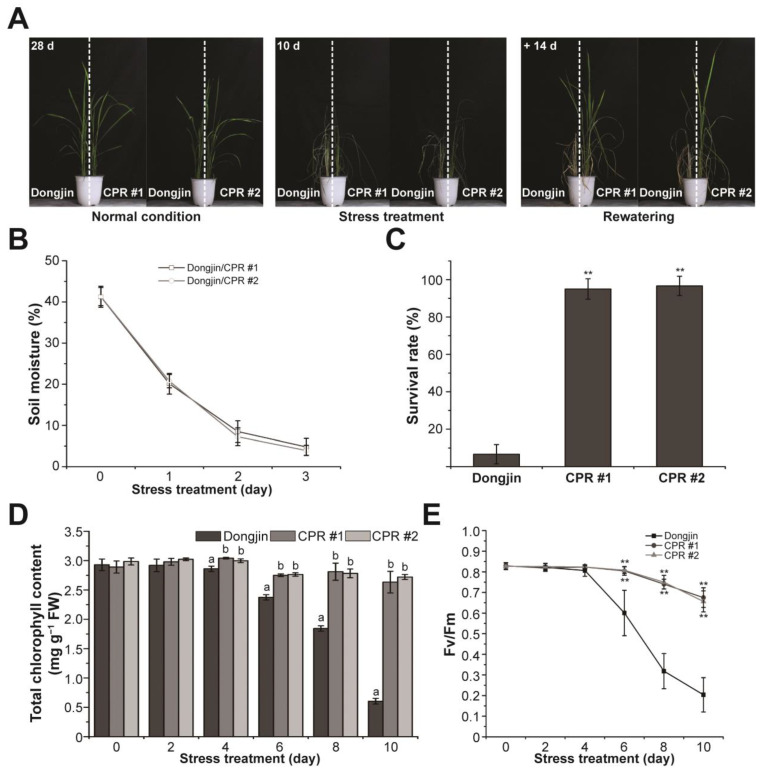
Caffeine production in CPR plants confers water-deficit stress tolerance. (**A**) Water-deficit stress tolerance phenotypes in CPR plants. Tolerance to water-deficit stress was assessed by growing CPR plants under normal conditions for 4 weeks, then withholding water for 10 days and rewatering for 14 days. (**B**) Measurement of soil moisture levels. All experiments were six biological replicates. Error bars represent the means ± SD (*n* = 6). (**C**) Survival of wild-type and CPR plants was measured 14 days after rewatering. All experiments were six biological replicates. Error bars represent the means ± SD (*n* = 6). Asterisks indicate statistically significant differences compared with wild-type (Dongjin) plants according to a Student’s *t*-test (** *p* < 0.01). (**D**) Accumulation of chlorophyll content in the wild-type and CPR plants during water-deficit stress treatment. All experiments were repeated three times with three biological replicates. Error bars represent the means ± SD (*n* = 9). Different letters above bars indicate statistically significant differences as determined by one-way analysis of variance (ANOVA), *p* < 0.01. (**E**) Chlorophyll fluorescence (Fv/Fm) of leaves during wild-type and CPR plants during water-deficit stress treatment. All experiments were repeated three times with four biological replicates. Error bars represent the means ± SD (*n* = 12), ** *p* < 0.01 (Student’s *t*-test).

The investigation of water-deficit tolerance was conducted using CPR plants (Figure 2A). Irrigation was withheld for 10 days on the four-leaf stage wild and CPR plants. To ensure that water-deficit stress was evenly distributed to both rice lines, we planted wild-type and CPR plants together in the same pot. Water-deficit stress treatment lasted 10 days, and soil moisture monitored for 3 days in each pot showed that each pot received the same amount of water-deficit stress (Figure 2B). In this period, leaves were withered in both wild-type and CPR plants, but it was more severe in the wild type (left side of the pot in the middle picture of Figure 2A). While the leaves of wild-type rice had turned yellow, the leaves of CPR had retained their green color to a significant extent even after withering. After 14 days of recovery with rewatering, the survival rate of CPR plants (CPR #1, 95%; CPR #2, 96.66%) was dramatically higher than that of wild-type plants (6.66%) (Figure 2C). During the 10-day period of water-deficit treatment, we conducted a comparative analysis of photosynthesis-related parameters between wild-type and CPR plants. The change in chlorophyll content was significantly different in wild-type and CPR plants. Chlorophyll content in wild-type plants distinguishably decreased after 6 days of withholding irradiation and was only about 1/6 after 10 days compared to CPR (Figure 2D). The Fv/Fm values, which represent the photochemical efficiency of photosystem (PS) II, exhibited a sharp decline in wild-type plants from the sixth day of the water-deficit stress treatment. In contrast, a slight decrease was observed in CPR plants (Figure 2E). These results suggest that caffeine in the CPR plants maintains a stable photosynthetic metabolism under water-deficit stress.

### 3.3. Endogenous Caffeine in CPR Plants Alters OsCPKs Expression

**Figure 3:**
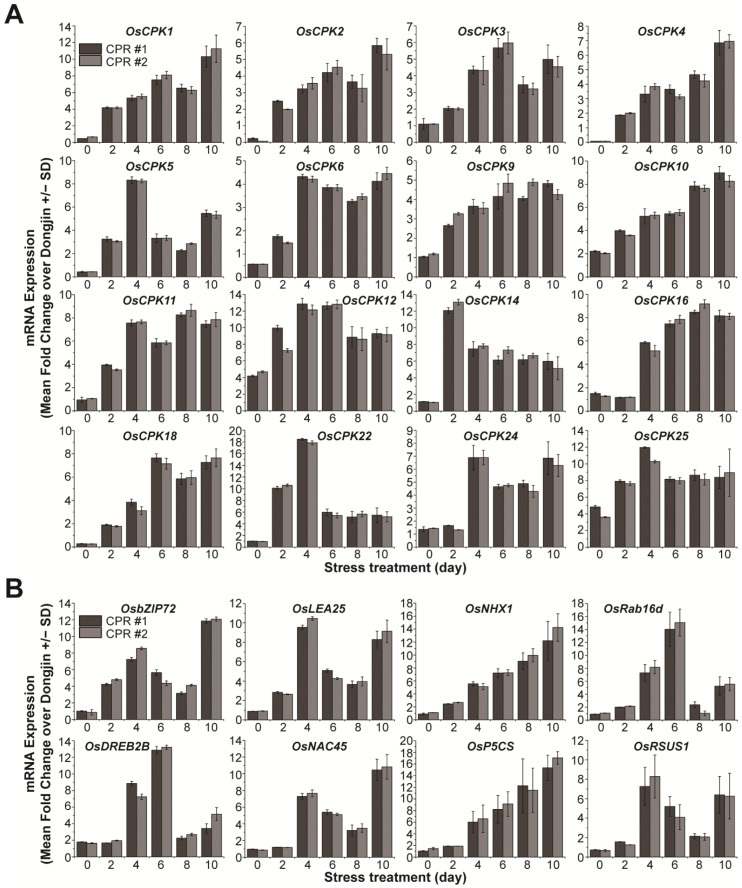
Gene expression analysis of *OsCPKs* and transcription factors under water-deficit stress treatment in wild-type and CPR plants. (**A**) Expression of *OsCPKs* gene in Dongjin and CPR plants under water-deficit stress treatment. (**B**) Expression of transcription factor genes involved in water-deficit tolerance under water deficit stress treatment. Total RNA was extracted from leaves of wild-type and CPR plants collected at 0, 1, 2, and 3 d after water-deficit stress treatment. The qRT-PCR was performed using specific primers for each gene. All experiments were repeated three times. Error bars represent the means ± SD (*n* = 3).

To further investigate the role of endogenous caffeine in water-deficit tolerance, we examined the expression of calcium signaling-related *OsCPKs* (Figure 3A). In the absence of water deficit, it was observed that seven *OsCPKs* exhibited upregulation of more than 2-fold in CPR plants compared to wild-type plants (Figure 1C). Under water-deficit stress, 16 *OsCPKs (OsCPK1*, LOC_Os01g43410; OsCPK2, LOC_Os01g59360; OsCPK3, LOC_Os01g61590; OsCPK4, LOC_Os02g03410; OsCPK5, LOC_Os02g46090, OsCPK6, LOC_Os02g58520; OsCPK9, LOC_Os03g48270; OsCPK11, LOC_Os03g57510; OsCPK14, LOC_Os05g41270; OsCPK16, LOC_Os05g39090; OsCPK18, LOC_Os07g22710; OsCPK22, LOC_Os09g33910; and OsCPK24, LOC_Os11g07040), including three genes (*OsCPK10*, *OsCPK12*, and *OsCPk25*) that have already increased their expression in CPR without water-deficit treatment, have increased more than 2-fold in CPR plants compared to wild type (Figure 3A). The expression of the remaining 15 *OsCPK* genes was either not different from the wild type in expression under water-deficit stress, downregulated, or upregulated, but the level of gene expression was not significant (Figure S2). We further examined the expression of 20 genes known to be involved in responses to water-deficit and osmotic stress (Figure 3B and Figure S3). In total, 8 (*OsbZIP72*, LOC_Os09g28310; *OsLEA25,* LOC_Os11g26570; *OsNHX1*, LOC_Os07g47100; *OsRab16d*, LOC_Os11g26780; *OsDREB2B*, LOC_Os05g27930; *OsNAC45*, LOC_Os11g03370; *OsP5CS*, LOC_Os05g38150; *OsRSUS1*, LOC_Os06g09450) of the 20 genes were expressed more than 2-fold in CPR plants compared to wild-type plants, while the remaining 12 genes were not significantly different from wild-type in expression under water-deficit stress (Figure S3). These eight transcription factor genes have been well studied and confirmed to be involved in tolerance to water-deficit and osmotic stress [71,72,73,74,75,76,77,78]. These results suggest that endogenous caffeine in CPR plants induces the expression of genes encoding CPKs and transcription factors that cause (or may trigger) resistance to water-deficit stress.

### 3.4. Endogenous Caffeine Suppresses Water-Deficit Stress-induced Production of H_2_O_2_ and MDA in Rice Plants

**Figure 4:**
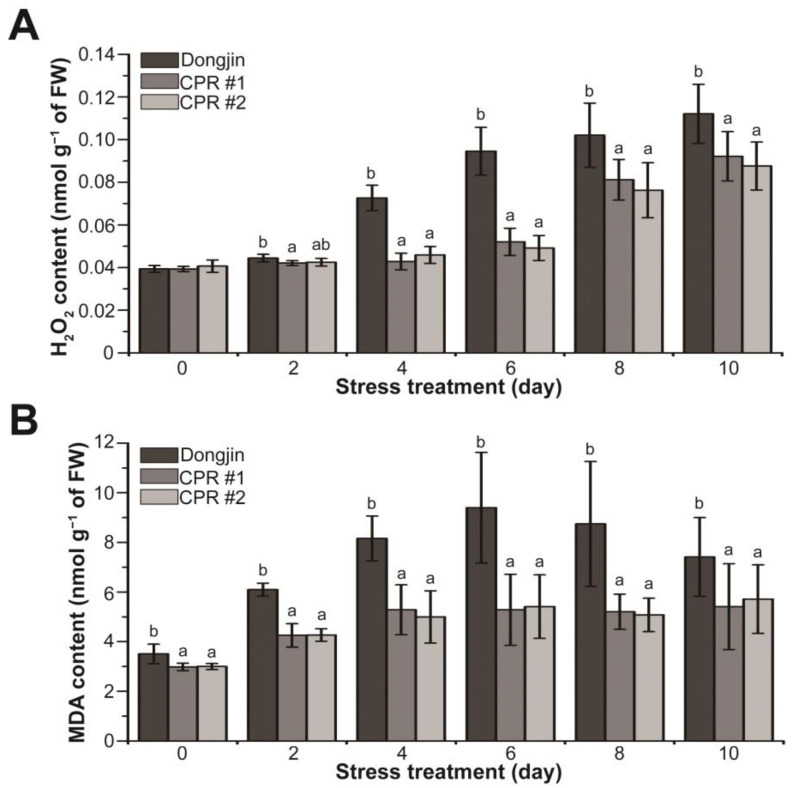
Analysis of changes in hydrogen peroxide and malondialdehyde levels during water-deficit stress treatment in wild-type and CPR plants. Quantification of H_2_O_2_ (**A**) and MDA levels (**B**) in wild-type and CPR plants under water-deficit stress. H_2_O_2_ and MDA were extracted from leaves of wild-type and CPR plants collected at 0, 2, 4, 6, 8, and 10 d after water-deficit stress treatment and quantified via spectrophotometry. All experiments were repeated three times with three biological replicates. Error bars represent the means ± SD (*n* = 9). Different letters above bars indicate statistically significant differences as determined by one-way analysis of variance (ANOVA), *p* < 0.05.

In plants, including rice, H_2_O_2_ and malondialdehyde (MDA), which is a substance produced by membrane lipids in response to reactive oxygen species (ROS) such as H_2_O_2_, are suggested to be increased by drought stress [79,80,81]. The alterations in H_2_O_2_ and MDA amounts were investigated in CPR subjected to water-deficit stress (Figure 4). Under normal growth conditions, the H_2_O_2_ levels of CPR plants and wild-type plants were alike, but the changes in H_2_O_2_ levels caused by water-deficit stress in the two plants were different (Figure 4A). During the water-deficit treatment period, both Dongjin and CPR plants exhibited a continuous increase in H_2_O_2_ levels. However, the extent of accumulation varied significantly. After four days of water-deficit treatment, H_2_O_2_ increased by 84.48% in Dongjin but only by 8.9% (CPR#1) and 12.87% (CPR#2) in CPR plants. Notably, ten days after the initiation of the water-deficit treatment, when the H_2_O_2_ content reached its peak, it had risen by 184.77% in Dongjin and by 134.37% (CPR#1) and 115.58% (CPR#2) in CPR plants (Figure 4A). The MDA levels in CPR plants were somewhat lower than in wild-type rice under normal conditions. It was 85.06% in CPR #1 and 85.52% in CPR #2 compared to Dongjin. The MDA levels in both Dongjin and CPR plants increased throughout the water-deficit stress treatment. Notably, in Dongjin, the MDA level reached its peak six days after the initiation of the water-deficit stress treatment (77.77% in CPR#1 and 73.6% in CPR#2 compared to Dongjin), after which it gradually decreased. In contrast, the CPR plants exhibited a relatively mild increase in MDA levels during the water-deficit treatment period and then maintained homeostasis (Figure 4B). These results suggest that endogenous caffeine in CPR plants contributes to reducing ROS production and lipid peroxidation caused by water-deficit stress.

### 3.5. Endogenous Caffeine Enhances Activity of Antioxidant Enzyme in CPR Plants under Water-Deficit Stress

**Figure 5:**
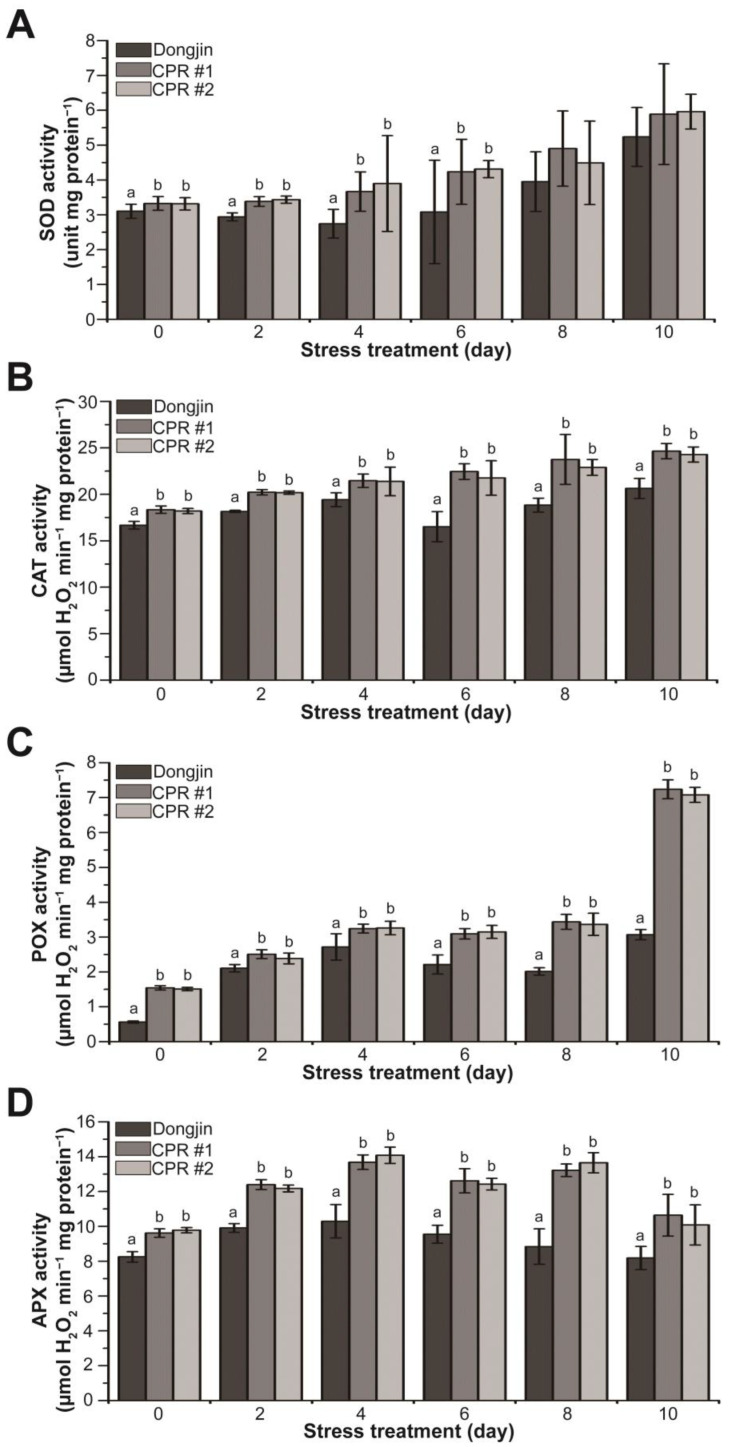
Analysis of activity changes in enzymatic antioxidants during water-deficit stress treatment in wild-type and CPR plants. (**A**) SOD activity of wild-type and CPR plants under water-deficit stress. (**B**) CAT activity of wild-type and CPR plants under water-deficit stress. (**C**) POX activity of wild-type and CPR plants under water-deficit stress. (**D**) APX activity of wild-type and CPR plants under water-deficit stress. Antioxidant enzymes were extracted from leaves of wild-type and CPR plants collected at 0, 2, 4, 6, 8, and 10 d after water-deficit stress treatment. The activities of the extracted antioxidant enzymes were measured by colorimetric spectrophotometry. All experiments were repeated three times with three biological replicates. Error bars represent the means ± SD (*n* = 9). Different letters above bars indicate statistically significant differences as determined via one-way analysis of variance (ANOVA), *p* < 0.05.

Based on the results shown in Figure 2E and Figure 4, we hypothesized that endogenous caffeine enhances the function of antioxidant enzymes. To validate this hypothesis, we measured the activity of antioxidant enzymes w/wo water-deficit stress. CPR plants had higher activities of SOD, CAT, POX, and APX compared to wild-type plants without water-deficit stress (Figure 5). In the case of POX, enzyme activity in CPR was nearly three times higher than that of Dongjin. During water deficit stress, SOD activity in Dongjin decreased to 11.56% after 4 days of water-deficit treatment, followed by a subsequent upward trend. In contrast, CPR plants showed a continuous increase in SOD activity throughout the entire period of water-deficit stress (Figure 5A). In Dongjin, CAT activity increased during 4 days of water-deficit stress treatment, decreased after 6 days, and then increased more. In contrast, CPR plants exhibited a continuous and moderate increase in CAT activity. The most substantial increase in CAT activity for Dongjin occurred at 4 days post-stress, rising by 16.46% compared to 0 days. In comparison, CPR plants showed increases of 16.97% (CPR#1) and 17.52% (CPR#2), respectively, during the same period (Figure 5B). The POX activity exhibited a gradual increase throughout the water-deficit stress period and showed a notable steepening ten days after the stress treatment. Notably, CPR plants displayed a 2.35- (CPR#1) and 2.30-fold (CPR#2) difference in POX activity compared to the wild-type plants, respectively (Figure 5C). Dongjin plants exhibited the highest APX activity four days after the water-deficit stress treatment, followed by a gradual decline. In contrast, CPR plants maintained high levels of APX activity until eight days after the water-deficit stress treatment. Notably, four days after the water-deficit stress treatment, when APX activity was at its peak, CPR plants displayed 32.91% (CPR#1) and 36.76% (CPR#2) higher activity compared to the wild type, respectively (Figure 5D). Overall, these results suggest that endogenous caffeine in CPR plants helps to mitigate the damage caused by high ROS due to water-deficit stress by increasing antioxidant enzyme activity.

### 3.6. Endogenous Caffeine Increases Non-Enzymatic Antioxidant Content of CPR Plants under Water-Deficit Stress

**Figure 6:**
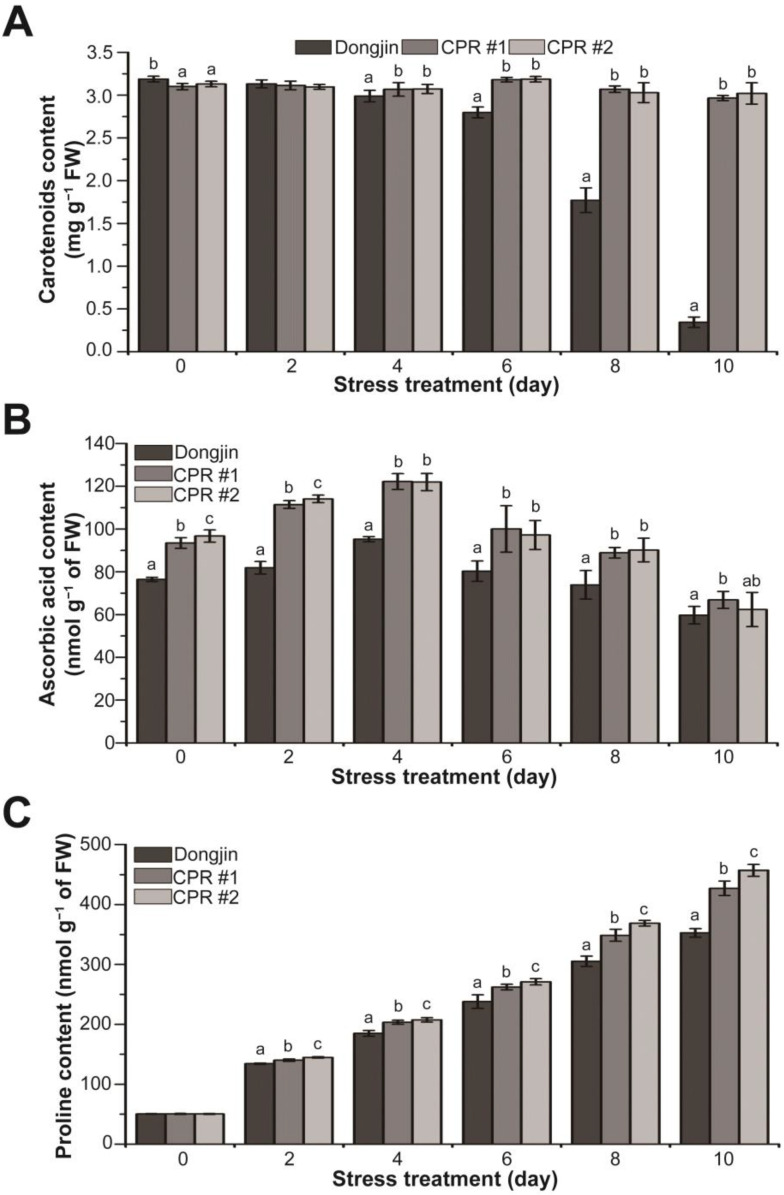
Analysis of changes in non-enzymatic antioxidants (carotenoids and ascorbic acid) and osmoprotectant (proline) contents during water-deficit stress treatment in wild-type and CPR plants. Quantification of carotenoids (**A**), ascorbic acid (**B**), and proline levels (**C**) in wild-type and CPR plants under water-deficit stress. The carotenoids, ascorbic acid, and proline were extracted from leaves of wild-type and CPR plants collected at 0, 2, 4, 6, 8, and 10 d after water-deficit stress treatment and quantified via spectrophotometry. All experiments were repeated three times with three biological replicates. Error bars represent the means ± SD (*n* = 9). Different letters above bars indicate statistically significant differences as determined via one-way analysis of variance (ANOVA), *p* < 0.05.

To further explore the role of endogenous caffeine in mitigating the damage caused by water-deficit stress, we analyzed changes in the levels of non-enzymatic antioxidants and osmoprotectants. Under normal growth conditions, CPR plants accumulated 1.8–2.7% less carotenoids than wild-type plants. Under water-deficit stress, carotenoid content declined dramatically in wild-type plants, whereas the content remained stable in CPR plants, with the difference in content between wild-type and CPR plants being 8.61 to 8.77-fold (Figure 6A). For ascorbic acid content, CPR plants accumulated 22–26% more than the wild-type plants under normal growth conditions. During the water-deficit stress treatment, there was a tendency for ascorbic acid accumulation to increase, particularly up to 4 days after the initiation of the stress treatment. CPR plants, in particular, accumulated 28.31% (CPR#1) and 28.07% (CPR#2) more ascorbic acid than the wild-type plants, respectively. Beginning four days after the water-deficit stress treatment, both the wild-type and CPR plants exhibited a trend of decreased ascorbic acid accumulation (Figure 6B). Under normal conditions, there was minimal difference in proline content between wild-type and CPR plants. The proline content in both wild-type and CPR plants exhibited an increase under the water-deficit stress treatment. However, during the water-deficit stress treatment, measurements taken at 2-day intervals revealed higher proline content in CPR plants, increasing by 4.28% (CPR#1), and 7.74% (CPR#2) two days after the stress treatment, 9.98% (CPR#1) and 12.16% (CPR#2) four days after the stress treatment, 10.21% (CPR#1) and 13.88% (CPR#2) six days after the stress treatment, 14.23% (CPR#1) and 20.81% (CPR#2) eight days after the stress treatment, and 21.11% (CPR#1) and 29.51% (CPR#2) in CPR plants ten days after the stress treatment, respectively (Figure 6C). These results indicate that endogenous caffeine plays a role in modulating the levels of non-enzymatic antioxidants and osmoprotectants, which are responsible for enhancing the tolerance of CPR plants to water-deficit stress.

## 4. Discussion

**Figure 7:**
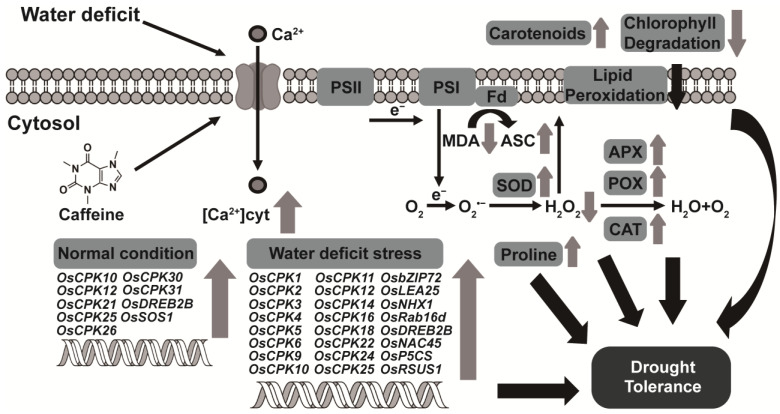
A model of endogenous caffeine-mediated tolerance regulation that induces water-deficit stress tolerance in CPR plants. Endogenous caffeine produced by CPR plants triggers an intracellular influx of calcium ions, which under normal growth conditions, upregulates the expression of OsCPK genes (*OsCPK10*, *OsCPK12*, *OsCPK21*, *OsCPK25*, *OsCPK26*, *OsCPK30*, *OsCPK31*) and water-deficit tolerance-related transcription factors (*OsDREB2B*, *OsSOS1*). Under water-deficit stress, these molecular level changes result in upregulation of OsCPK genes (*OsCPK1*, *OsCPK2*, *OsCPK3*, *OsCPK4*, *OsCPK5*, *OsCPK6*, *OsCPK9*, *OsCPK10*, *OsCPK11*, *OsCPK12*, *OsCPK14*, *OsCPK16*, *OsCPK18*, *OsCPK22*, *OsCPK24*, *OsCPK25*) and water-deficit tolerance-related transcription factors (*OsbZIP72*, *OsLEA25*, *OsNHX1*, *OsRab16d*, *OsDREB2B*, *OsNAC45*, *OsP5CS*, *OsRSUS1*), leading to biochemical level changes such as reduced accumulation of ROS and MDA, increased activity of antioxidant enzymes (SOD, CAT, POX, APX), and increased accumulation of non-enzymatic antioxidants (carotenoids, ASA, proline). These biochemical changes then lead to physiological changes such as inhibition of lipid peroxidation, reduction of chlorophyll degradation, and protection of photosynthetic machinery, ultimately resulting in acquired tolerance to water-deficit stress. Abbreviations: APX, ascorbate peroxidase; CAT, catalase; ASC, ascorbic acid; Fd, ferredoxin; MDA, malondialdehyde; POX, peroxidase; PSI/II, photosystem I/II; SOD, superoxide dismutase.

Plants must constantly adapt to a variety of challenges, including biotic and abiotic stresses. The outcome of these interactions with stresses is crucial for determining a plant’s survival. Especially in cereal plants, rapid and adequate responses to mitigate biotic and/or abiotic stresses are very important for crop productivity. It is now estimated that nearly 800 million people face hunger [82]; minimizing the loss in crop products due to stresses is, therefore, one of the effective ways to solve this food problem of the world. In order to achieve this objective, a lot of effort is dedicated to the identification of diverse secondary metabolites originating from plants as well as the investigation of their function in relation to stress resistance. Several studies, including our previous report, have shown that caffeine, a well-known secondary metabolite, confers biotic stress resistance directly or indirectly [24,25,26,27,83]. Caffeine acts directly as an antimicrobial and repellent and indirectly as a triggering agent for resistance signaling. Caffeine increases salicylic acid levels via calcium signaling, making plants more resistant to variable biotic stresses [27]. However, the knowledge of the role of caffeine in abiotic stress tolerance is very limited. Recently, exogenous caffeine application to spinach has been shown to improve resistance to cadmium toxicity [84]. We now report that this study provides evidence that caffeine also has a positive effect on water-deficit tolerance in rice. Wild-type rice, Dongjin, exhibited a very poor survival rate due to the disorders of physiological and biochemical metabolism caused by the water-deficit stress (Figure 2, Figure 3, Figure 4, Figure 5 and Figure 6). On the other hand, CPR under water-deficit stress can express more water-deficit tolerance-related genes such as particular *CPKs* and transcription factors, has higher activities of antioxidant enzymes, and has increased non-enzymatic antioxidant accumulation (Figure 3, Figure 5 and Figure 6). These alterations are able to alleviate water-deficit stress-induced damage in rice plants, as shown in our working model (Figure 7). All the changes shown in this present study are most likely due to Ca^2+^ signaling triggered by caffeine. Although we did not repeat the investigation of the change in intracellular Ca^2+^ concentration in this study, the CPR has much a higher Ca^2+^ concentration in cytosol than Dongjin [27].

Previous studies have shown that the amount of caffeine synthesized in CPR plants depends on the amount of its precursor, xanthosine, and that HPLC-detectable caffeine is synthesized in CPR plants from the two-leaf stage onward [27]. Caffeine, as a type of methylxanthine, acts as a non-selective inhibitor of phosphodiesterase [85]. The enzyme catalyzes a hydrolysis reaction from 3′, 5′-cAMP/3′, 5′-cGMP to AMP/GMP [86], thereby causing an abnormal accumulation of cAMP/cGMP in CPR plants [27]. High levels of cAMP/cGMP, which are involved in signal transduction, bind to cyclic nucleotide-gated channels in the cell membrane and activate the channels [87], leading to Ca^2+^ accumulation in the cytosol [27]. The high intracellular concentration of Ca^2+^ is recognized by calcium-binding proteins that act as calcium sensors, initiating an immediate calcium-mediated signal transduction in plants [40]. Ca^2+^ signaling has been known to play a broad and diverse role in plants, including the growth, development, stress perception, and regulation of cell responses [40,88]. In particular, a specific CPK plays a critical role in the Ca^2+^ signaling pathway that mediates responses to biotic and abiotic stresses by regulating important processes such as ROS generation and hypersensitive reactions [40,89]. We analyzed the transcript levels of 31 *OsCPK* genes to identify alterations in Ca^2+^ signaling induced by endogenous caffeine at the four-leaf stage. We found that the expression levels of seven *OsCPK* genes were significantly increased in CPR plants under normal growth conditions (Figure 1). Although two *OsCPKs* (*OsCPK30* and *OsCPK31*) have not been mentioned in relation to abiotic stress tolerance, the five CPKs are known to be involved in abiotic stress resistance. The overexpression of *OsCPK10* resulted in decreased accumulation of H_2_O_2_ and MDA levels, reducing lipid peroxidation and ultimately demonstrating resistance to drought stress by phosphorylating CAT-A [66]. The overexpression of *OsCPK12* increased the expression levels of *OsAPX2* and *OsAPX8*, which encode APX, resulting in increased ROS detoxification, thus demonstrating resistance to salt stress [67]. The overexpression of *OsCPK21* increased the expression levels of stress-related transcription factors such as *OsP5CS*, *OsLEA3*, *OsNAC6*, *OsbZIP23*, *OsNHX1*, and *OsSOS1*, demonstrating resistance to salt stress [68]. The detailed function of water-deficit tolerance remains unclear, *OsCPK26* is a gene that is upregulated in drought tolerance isogenic lines under water-deficient conditions [90]. Under water-deficit conditions, the expression of many CPKs in CPR plants was surprisingly enhanced. The expression of three (*OsCPK10*, *OsCPK12*, and *OsCPK25*) of the seven *OsCPKs* that were already increased in CPR without water-deficit treatment was further enhanced with water-deficit stress. However, the expression of the rest of the four *OsCPKs* (*OsCPK21*, *OsCPK26*, *OsCPK30*, and *OsCPK31*) declined with water-deficit treatment and did not show a big difference with those of the wild type at three days after water-deficit treatment. In addition to the 3 *OsCPKs*, the expression of the 13 *OsCPKs* (*OsCPK1*, *OsCPK2*, *OsCPK3*, *OsCPK4*, *OsCPK5*, *OsCPK6*, *OsCPK9*, *OsCPK11*, *OsCPK14*, *OsCPK16*, *OsCPK18*, *OsCPK22*, and *OsCPK24*) markedly enhanced compared to wild-type rice plants with water-deficit treatment (Figure 2A). Some of these genes have been reported to play a role in response to abiotic stress, especially drought, but five of them (*OsCPK3*, *OsCPK5*, *OsCPK16*, *OsCPK18*, and *OsCPK24*) have not yet. The *OsCPK1* gene is known to be activated by sucrose and involved in drought tolerance mechanism [91]. CPK1 negatively regulates GA biosynthesis and activates 14-3-3 protein expression to prevent damage by drought stress in the germination stage. To date, there have been no reports investigating the role of CPK2 in rice and its connection to drought stress. However, it is worth noting that barley CPK2 has been found to function as a negative regulator of the drought stress response [92]. *OsCPK4* expression is induced by salt, drought, and ABA. The overexpression of *OsCPK4* confers significant tolerance to salt and drought stress via the protection of cellular membranes from stress-induced oxidative damage [93]. OsCPK6 is a gene that is upregulated under drought stress [70]. OsCPK9 plays a positive role in drought stress tolerance and spikelet fertility. OsCPK9 enhances stomatal closure and improves osmotic adjustment ability in plants [94]. The investigation of wild-type and heterozygous mutants of *OsCPK11* suggested that *OsCPK11* is likely involved in salt, cold, and drought stress signaling [95]. *OsCPK14* interacts with *OsDi19-4*, one of the drought-induced 19 transcription factor family, with an overexpression that enhances drought tolerance in *Arabidopsis* [96]. OsCPK14 is, therefore, suggested as an indirect regulator of drought stress. Using knockout and overexpression mutants, the role of OsCPK24 as a positive regulator of cold stress has been elucidated [97]. In addition, the expression of the eight transcription factors related to abiotic stress resistance was also altered in CPR plants (Figure 3B). One member of the basic leucine zipper (bZIP) family, *OsbZIP72*, is hypersensitive to ABA and plays a positive role in drought stress resistance by increasing the expression levels of late embryogenesis abundant (LEA) genes, which are ABA-responsive genes that enhance drought tolerance [72]. The exogenous application of melatonin confers salt stress tolerance in rice plants, and under these conditions, *OsLEA25* is upregulated as a responsive gene [71]. The overexpression of rice Na^+^/H^+^ exchanger 1 (*OsNHX1*) enhances resistance to both drought and salt stress [73,74]. *OsRab16d* is associated with ABA signaling pathways and increases stress tolerance in response to drought and salt stress [98]. The overexpression of rice dehydration-responsive element 2B (*OsDREB2B*) confers strong drought tolerance [76], and the overexpression of *OsDREB2B* in Arabidopsis increases resistance to drought and heat-shock stress [99]. The overexpression of rice *OsNAC45* mediates POX activity and plays an important role in drought and salt stress tolerance by responding to ABA [75,100]. The *OsPC5S* gene contributes to drought stress tolerance by enhancing proline accumulation and the activity of antioxidant enzymes [78]. The expression of rice sucrose synthase1 (*OsRSUS1*) is upregulated as a responsive gene to enhance drought stress tolerance [101,102]. Although we are not able to explain how water-deficit signals mediated and distributed by particular CPKs are delivered to these transcription factors in the signaling pathways of rice, it is clear that caffeine synthesized in CPR plants triggers significant changes that can cope with abiotic stresses including water deficit.

Drought stress accelerates the aging process of plant leaves, causing a rapid breakdown of chlorophyll and a reduction in photosynthesis that significantly impacts the lifespan of plants. This process leads to an excessive accumulation of ROS within the plant, which can cause oxidative damage to cell membranes, essential enzymes, proteins, nucleic acids, and more [103,104,105,106,107]. Like the leaves of the Dongjin, the leaves of the CPR plants wilted from the water-deficit treatment but remained greener than the wild-type plants and recovered nearly 100% with watering (Figure 2). This is because CPR plants excellently retain chlorophyll content, photosystem II photochemical efficiency (Figure 2), H_2_O_2_, and MDA content under water-deficit stress (Figure 4), whereas wild types do not. These caffeine-induced changes are attributed to protecting the CPR plants from oxidative damage caused by water-deficit stress.

Plants exhibit homeostasis by effectively regulating the amount of reactive oxygen species (ROS) to maintain optimal levels. However, abiotic stress disrupts regulatory mechanisms, leading to excessive ROS accumulation and oxidative stress [108]. Meanwhile, plants have well-developed antioxidant defense systems, including enzymatic and non-enzymatic antioxidants [108,109]. According to a recent report, the application of exogenous caffeine enhances the capacity of the antioxidant defense system. Exogenous caffeine reduces cadmium stress-induced ROS accumulation by increasing the enzymatic activities of SOD, CAT, and POX and increasing ascorbic acid content. These biochemical changes induced by caffeine conferred resistance to cadmium in plants [84]. In CPR plants, endogenous caffeine activates the antioxidant defense system. Under no water-deficit stress, all four enzymes (SOD, CAT, POX, and APX) scavenging ROS have higher activity than Dongjin, and the activity significantly increased by water-deficit treatment, except SOD (Figure 5). While SOD activity persisted in CPR plants, it diminished in wild-type rice under water-deficit stress (Figure 5A). These results suggest that SOD functions normally under water-deficit stress in CPR but not in wild-type plants. Non-enzymatic antioxidants in CPR plants, such as carotenoids, ascorbic acid, and proline, also showed significant differences in content compared to wild-type rice (Figure 6). Carotenoids are pigments that give fruits and vegetables their color. They have antioxidant properties that can protect cells from free radical damage by donating electrons to free radicals or scavenging singlet oxygen, a highly reactive form of oxygen [110]. The antioxidant properties of ascorbic acid are attributed to its ability to donate hydrogen atoms to lipid radicals, neutralize singlet oxygen, and eliminate molecular oxygen [111]. Proline accumulates in plant cells under stress conditions, allowing the maintenance of plant macromolecules and structure from stress damage via ROS detoxification. Additionally, it acts as an effective osmo-protectant that maintains cell turgor pressure balance, increasing plant resistance to osmotic stress [81,104,112]. Proline accumulation and enhanced drought stress tolerance are closely associated with the *OsP5CS* gene product [113]. *OsP5CS* encodes an enzyme called Δ1-pyrroline-5-carboxylate synthetase, which is involved in the biosynthesis of proline, an amino acid that helps plants cope with drought and salt stress [114]. In CPR plants, the expression of *OsP3CS* also increased with water-deficit treatment (Figure 3). Taken together, caffeine uses both enzymatic and non-enzymatic antioxidants present in plants to strengthen antioxidant defense mechanisms and lessen the oxidative damage water-deficit stress causes to rice.

We previously reported that caffeine as a defense inducer confers multi-resistance against variable biotic stresses, including bacterial, fungal, and insect pathogens, in rice without fitness cost [27]. Caffeine directly functions as an antibacterial and insect repellent agent and as a signal molecule to trigger Ca^2+^-mediated defense responses. In the present study, we report the novel discovery of the additional properties of caffeine as an enhancer conferring tolerance to abiotic stress in rice plants. The rice plants producing caffeine exhibited significant resistance to water-deficit stress and demonstrated a remarkable survival rate upon re-irrigation following a severe water-deficit treatment. At the molecular level, as shown in the working model (Figure 7), there were notable disparities in the expression patterns of *OsCPKs* and some genes implicated in the plant abiotic stress response between CPR and wild-type rice. CPR also robustly maintains photosynthetic capacity under water-deficit stress. Furthermore, the activity of ROS-detoxifying enzymes (SOD, CAT, POX, and APX) and the production of non-enzymatic antioxidants (carotenoid, ascorbic acid, and proline) are enhanced in CPR, resulting in tolerance to water deficit-induced oxidative damage. Although this study could not provide evidence of how the Ca^2+^ signals decoded via CPKs are delivered to the response molecules, these biochemical changes induced by endogenous caffeine are sufficient to obtain strong resistance in rice plants. In this study, following on from our previous report [27], we suggest again how versatile and effective caffeine is as a priming agent and confidence its application in agriculture will contribute to exceptional rice productivity.

## 5. Conclusions

In conclusion, the endogenous production of caffeine in CPR plants resulted in a stress-ready state, enhancing their tolerance to water-deficit stress, a significant threat to growth and survival. The stable production of caffeine in transgenic CPR plants over generations led to the upregulation of seven OsCPK genes (*OsCPK10*, *OsCPK12*, *OsCPK21*, *OsCPK25*, *OsCPK26*, *OsCPK30*, *OsCPK31*) and two stress tolerance-related transcription factor genes (*OsDREB2B*, *OsSOS1*) even under normal growth conditions. These molecular changes led to biochemical changes, such as decreased MDA accumulation, increased antioxidant enzyme activity (SOD, CAT, POX, APX), and accumulation of the non-enzymatic antioxidant ascorbic acid, equipping CPR plants with defense against water-deficit stress. Under water-deficit stress, rice suffers from oxidative stress that accelerates chlorophyll degradation and reduces photosynthesis due to increased ROS and MDA accumulation. However, endogenous caffeine produced in CPR plants upregulated the expression of 16 OsCPK genes (*OsCPK1*, *OsCPK2*, *OsCPK3*, *OsCPK4*, *OsCPK5*, *OsCPK6*, *OsCPK9*, *OsCPK10*, *OsCPK11*, *OsCPK12*, *OsCPK14*, *OsCPK16*, *OsCPK18*, *OsCPK22*, *OsCPK24*, *OsCPK25*) and 8 stress tolerance-related transcription factor genes (*OsbZIP72*, *OsLEA25*, *OsNHX1*, *OsRab16d*, *OsDREB2B*, *OsNAC45*, *OsP5CS*, *OsRSUS1*), leading to increased activities of antioxidant enzymes (SOD, CAT, POX, APX) and reduced ROS and MDA accumulation via non-enzymatic antioxidant (carotenoids, ascorbic acid, proline) accumulation. These molecular and biochemical changes resulted in significant tolerance to water-deficit stress, ultimately inhibiting chlorophyll degradation and protecting photosynthetic machinery under water-deficit stress. Our results provide insights into metabolic engineering approaches using plant secondary metabolites for stress tolerance and offer a potential avenue for the discovery of novel molecular breeding sources.

## Data Availability

The data are contained within this article.

## Supplementary Materials

The following supporting information can be downloaded at: https://www.mdpi.com/article/10.3390/antiox12111984/s1, Table S1: Primers used for RT-PCR and qRT-PCR analysis. Figure S1: Gene expression analysis of *OsCPKs* in wild-type and CPR plants at the four-leaf stage under normal conditions. Figure S2: Gene expression analysis of *OsCPKs* under water-deficit stress treatment in wild-type and CPR plants. Figure S3: Gene expression analysis of transcription factors under water-deficit stress treatment in wild-type and CPR plants.
