# Supplementary figures and images for: Caffeine Produced in Rice Plants Provides Tolerance to Water-Deficit Stress
Source: Antioxidants (Basel). 2023 Nov 8;12(11):1984. doi: 10.3390/antiox12111984 (PMC10669911; doi:10.3390/antiox12111984)

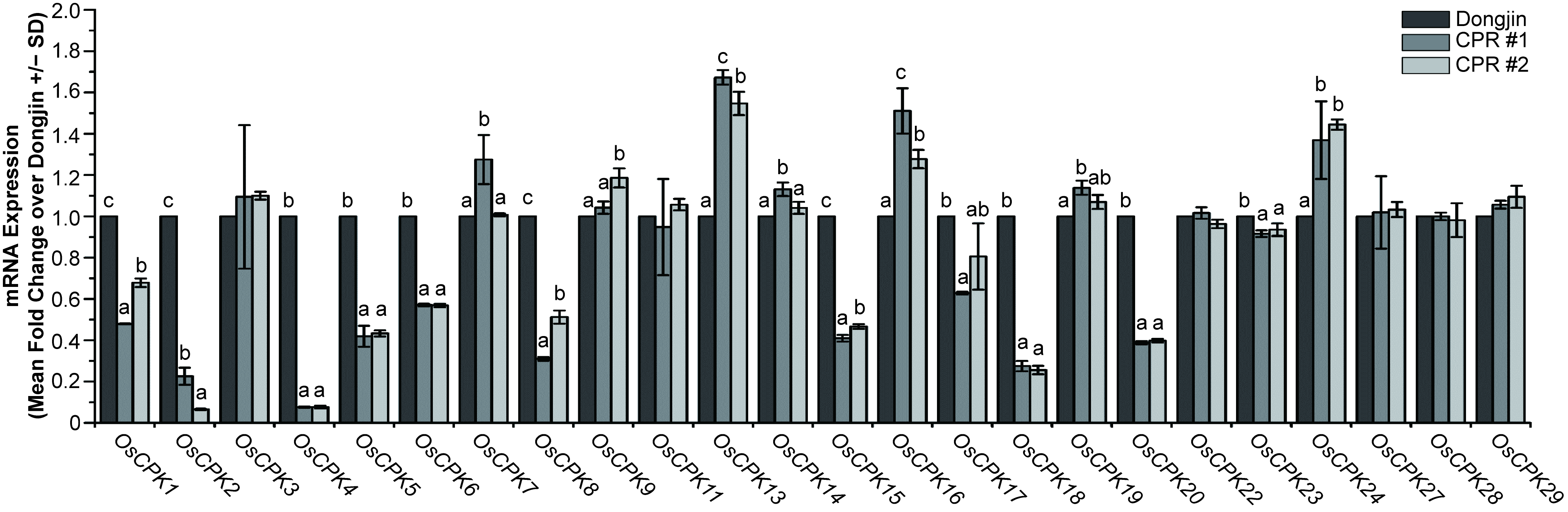

Supplement: Supplementary file 1 [file antioxidants-12-01984-s001.zip › Supplementary_Figure S1_r.tif]

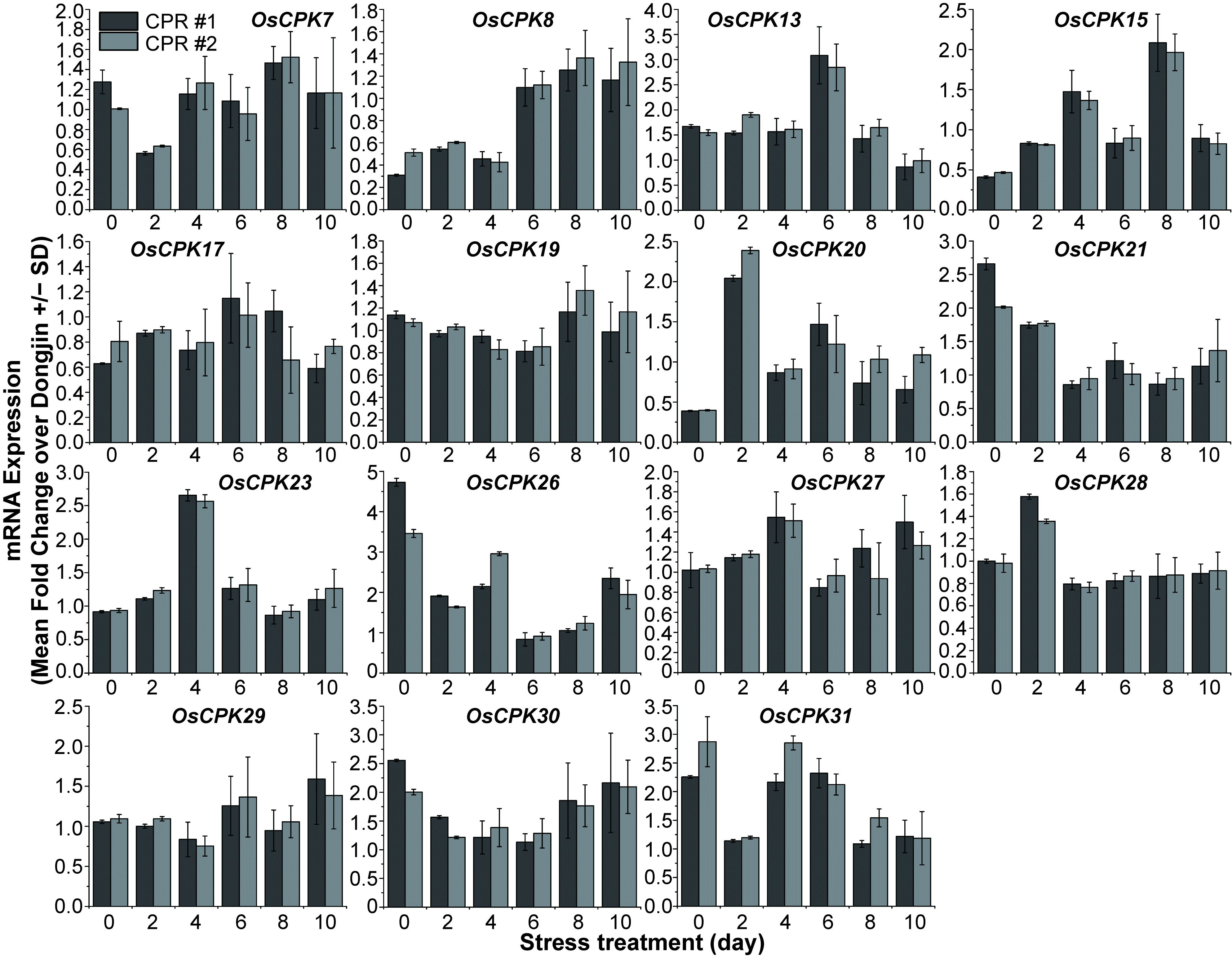

Supplement: Supplementary file 1 [file antioxidants-12-01984-s001.zip › Supplementary_Figure S2_r2.tif]

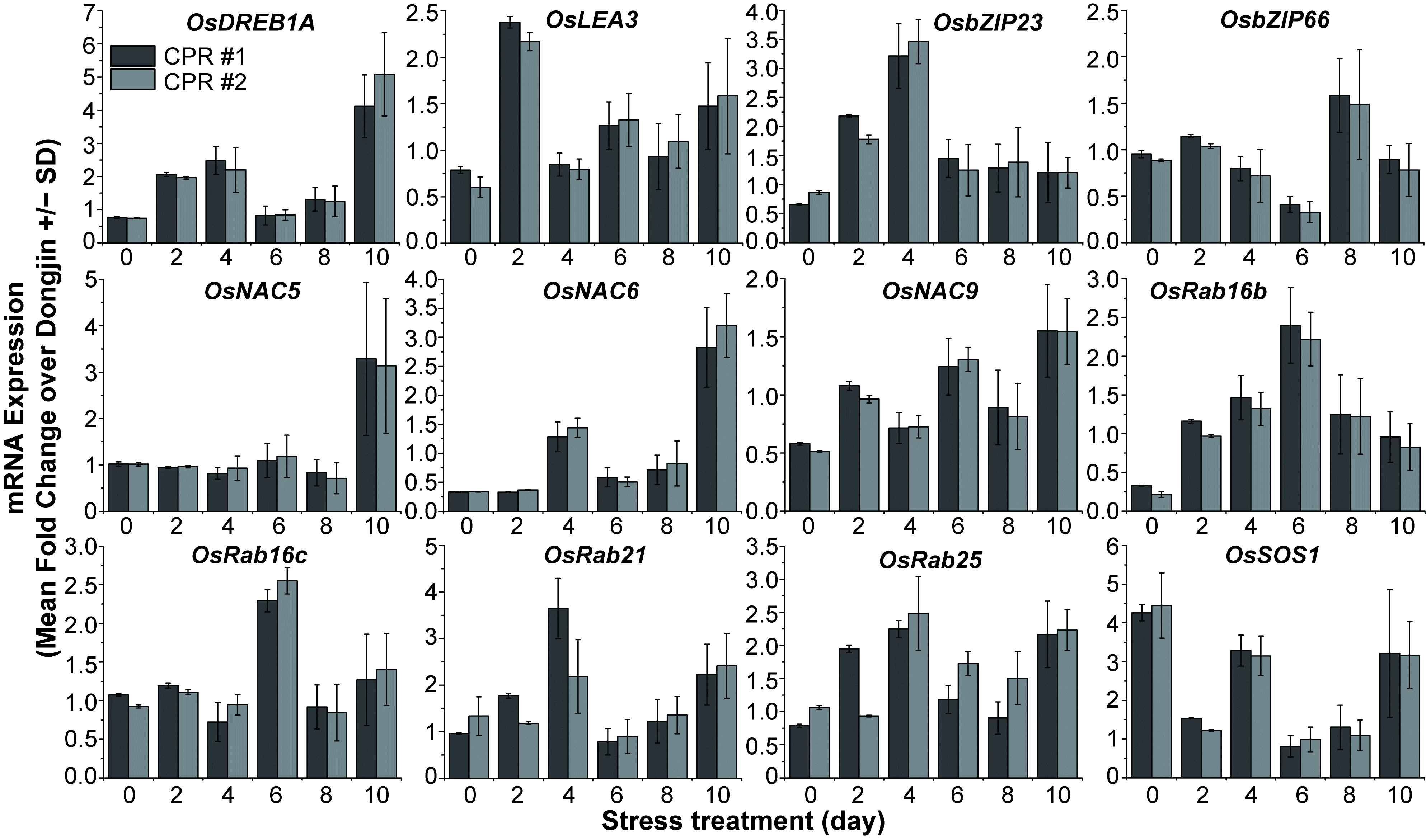

Supplement: Supplementary file 1 [file antioxidants-12-01984-s001.zip › Supplementary_Figure S3_r2.tif]
